# *Mycoplasma hominis* and *Mycoplasma genitalium* in the Vaginal Microbiota and Persistent High-Risk Human Papillomavirus Infection

**DOI:** 10.3389/fpubh.2017.00140

**Published:** 2017-06-26

**Authors:** Sally N. Adebamowo, Bing Ma, Davide Zella, Ayotunde Famooto, Jacques Ravel, Clement Adebamowo, ACCME Research Group

**Affiliations:** ^1^Department of Epidemiology and Public Health, University of Maryland School of Medicine, Baltimore, MD, United States; ^2^University of Maryland Comprehensive Cancer Center, University of Maryland School of Medicine, Baltimore, MD, United States; ^3^Institute for Genome Sciences, University of Maryland, Baltimore, MD, United States; ^4^Department of Microbiology and Immunology, University of Maryland School of Medicine, Baltimore, MD, United States; ^5^Institute of Human Virology, University of Maryland School of Medicine, Baltimore, MD, United States; ^6^Institute of Research Virology Nigeria, Abuja, Nigeria

**Keywords:** *Mycoplasma hominis*, *Mycoplasma genitalium*, vaginal microbiota, persistent high-risk HPV, human papillomavirus, Nigeria

## Abstract

**Background:**

Recent studies have suggested that the vaginal microenvironment plays a role in persistence of high-risk human papillomavirus (hrHPV) infection and thus cervical carcinogenesis. Furthermore, it has been shown that some mycoplasmas are efficient methylators and may facilitate carcinogenesis through methylation of hrHPV and cervical somatic cells. We examined associations between prevalence and persistence of *Mycoplasma* spp. in the vaginal microbiota, and prevalent as well as persistent hrHPV infections.

**Methods:**

We examined 194 Nigerian women who were tested for hrHPV infection using SPF_25_/LiPA_10_ and we identified *Mycoplasma genitalium* and *Mycoplasma hominis* in their vaginal microbiota established by sequencing the V3–V4 hypervariable regions of the 16S rRNA gene. We defined the prevalence of *M. genitalium, M. hominis*, and hrHPV based on positive result of baseline tests, while persistence was defined as positive results from two consecutive tests. We used exact logistic regression models to estimate associations between *Mycoplasma* spp. and hrHPV infections.

**Results:**

The mean (SD) age of the study participants was 38 (8) years, 71% were HIV positive, 30% *M. genitalium* positive, 45% *M. hominis* positive, and 40% hrHPV positive at baseline. At follow-up, 16% of the women remained positive for *M. genitalium*, 30% for *M. hominis*, and 31% for hrHPV. There was a significant association between persistent *M. hominis* and persistent hrHPV (OR 8.78, 95% CI 1.49–51.6, *p* 0.01). Women who were positive for HIV and had persistent *M. hominis* had threefold increase in the odds of having persistent hrHPV infection (OR 3.28, 95% CI 1.31–8.74, *p* 0.008), compared to women who were negative for both.

**Conclusion:**

We found significant association between persistent *M. hominis* in the vaginal microbiota and persistent hrHPV in this study, but we could not rule out reverse causation. Our findings need to be replicated in larger, longitudinal studies and if confirmed, could have important diagnostic and therapeutic implications.

## Introduction

Globally, cervical cancer is the fourth most common cancer in women, with an estimated 528,000 new cancers in 2012 (1). It is one of the two commonest cancers in low- and middle-income countries where the mortality rate from cervical cancer is also very high. Persistent high-risk human papillomavirus (hrHPV) infection is a necessary but not sufficient cause for cervical intraepithelial neoplasm grades 2/3 and cervical cancer (2). While most hrHPV infections are cleared within 2 years, it persists in about 12% of women who remain at an elevated risk for the development of cervical cancer (3). Although the reasons for this variable natural history continue to be studied, it is generally accepted that several cofactors are important for the development of cervical cancer in hrHPV-infected women (2, 4). Identification of these cofactors will improve understanding of cervical carcinogenesis and identify new opportunities for prevention and treatment of cervical cancer.

Sexually transmitted genital pathogens such as HIV, herpes simplex virus, *Neisseria gonorrhea, Chlamydia trachomatis, Gardnerella vaginalis, Trichomonas vaginalis, Ureaplasma urealyticum, Ureaplasma parvum*, and *Treponema pallidum* have been identified as possible cofactors of persistent hrHPV infection in cervical carcinogenesis. However, the results from previous studies have been inconsistent (5–11). Several pathogens, particularly those capable of establishing persistent infections can affect cellular apoptotic pathways and potentially facilitate abnormal cell growth (12). Some *Mycoplasma* spp. have been shown to be capable of persistent infections and induction of somatic cellular chromosomal alterations that lead to neoplastic transformation in several tissues (13–19). Few studies have examined the relationship between some *Mycoplasma* spp. and risk of hrHPV infections or CIN2+ in women (6, 8–10). Some of these studies showed no associations between *Mycoplasma genitalium* and hrHPV infections (8, 10), non-significant associations between *M. genitalium, Mycoplasma hominis*, and hrHPV infections (9), or significant associations between *M. genitalium, M. hominis*, and hrHPV infections (6). To date, no study has examined the association between prevalence or persistence of *Mycoplasma* spp. and persistent hrHPV infections.

In this study, we examined associations between prevalent and persistent *M. genitalium* and *M. hominis*, and hrHPV infections in a cohort of Nigerian women.

## Materials and Methods

### Study Population

We studied 1,020 women who were enrolled in a study of HPV infection and cervical cancer at National Hospital, Abuja and University of Abuja Teaching Hospital, Nigeria, between 2012 and 2014. All the study participants were 18 years or older, had a history of vaginal sexual intercourse, were not currently pregnant and had no history of hysterectomy. Trained nurses collected data on socio-demographic characteristics, sexual and reproductive history, and self-reported HIV status of the participants. We confirmed self-reported HIV status of participants from the hospitals’ medical records. The nurses collected venous blood samples and performed pelvic examinations on all study participants at each study visit. We used Elution swab systems (Copan, Italy) to collect mid-vaginal swabs and cervical brushes to collect exfoliated cervical cells, which were inserted in 1 ml Amies’ transport media (Copan). Participants were asked to return for follow-up after 6 months, at which time, the history, physical examinations, and samples’ collections were repeated. All samples were stored at −80°C, until processing for further analysis. In this analysis, we included 194 women who had data on mycoplasma and HPV.

### HPV Detection by SPF_10_/LiPA_25_

We extracted DNA from cervical exfoliated cells as previously described (5). Samples were tested for the presence of HPV DNA by hybridization of SPF_10_ amplimers to a mixture of general HPV probes recognizing a broad range of HPV genotypes in a microtiter plate format, as previously described (20). All samples determined to be HPV DNA positive by SPF_10_ DNA Enzyme Immunoassay (DEIA) were genotyped using the LiPA_25_ version 1. The LiPA_25_ assay provides type-specific information for 25 different HPV genotypes simultaneously and identifies infection by one or more of 13 hrHPV genotypes: 16, 18, 31, 33, 35, 39, 45, 51, 52, 56, 58, 59, and 68 (21, 22). We defined hrHPV infection as prevalent if at least one hrHPV genotype was detected by the SPF_10_/LiPA_25_ test in a sample provided at the baseline visit; and persistent if at least one hrHPV genotype was detected by the SPF_10_/LiPA_25_ test in samples provided at the baseline and follow-up visits.

### Sequencing and Analysis of Barcoded 16S rRNA Gene Amplicons

We extracted genomic DNA from mid-vaginal swabs as previously described (23). Dual barcode system fusion primers 338 and 806R were used for PCR amplification of the V3–V4 hypervariable regions of the 16S rRNA gene as previously described (24). Both positive and negative controls for DNA extraction and PCR amplification were included. Amplicons were pooled and sequenced on an Illumina MiSeq instrument using the 300 bp paired-end protocol, at the Institute for Genome Sciences, University of Maryland School of Medicine. Raw reads were preprocessed to remove the first 3 and last 3 bases if their Phred score was lower than 3. Read ends were trimmed if the average Phred quality score of 4 consecutive bases was below 15. Paired reads were retained if their length was at least 75% of their original length after trimming. QIIME (v1.8.0) (25) was used to perform quality control of the sequence reads. Reads were assembled using Fast Length Adjustment of Short reads (FLASH) (26), with an overlap of ~90 bp on average. Assembled reads were de-multiplexed by binning sequences with the same dual barcode. Both *de novo* and reference-based chimera detection were conducted in UCHIME (v5.1) using Greengenes database of 16S rRNA gene sequences (Aug 2013) as a reference (27, 28). The processed 16S rRNA gene amplicon sequences were assigned to genera and species, using PECAN that uses fifth-order Markov Chain model for precise species-level assignments and a pre-compiled database that contains all known microbes in the vaginal microbiota. Ward linkage clustering was used to cluster samples based on their Jensen–Shannon distance calculated in vegan package in R (29). *M. genitalium* and *M. hominis* were defined as prevalent if at least one read of the spp. was detected by sequencing, in a sample provided at the baseline visit and persistent if at least one read of the spp. was detected by sequencing, in samples provided at the baseline and follow-up visits.

### Statistical Analysis

We managed participants’ data using REDCap electronic database, hosted at the Institute of Human Virology Nigeria (30, 31). While all of these women had complete data on *M. hominis* and HPV at both visits, 27% (52/194) did not have data on *M. genitalium* at baseline and were excluded from the *M. genitalium* analysis. We examined differences in the distribution of continuous variables between groups using *t*-tests and used χ^2^ and Fisher’s exact tests for categorical variables. We used exact logistic regression (32, 33) to examine the associations between *M. genitalium* and hrHPV infections, and *M. hominis* and hrHPV infections. We considered characteristics that were significant at the *p* < 0.20 level in age adjusted analyses for inclusion in multivariate models. All analyses were performed using SAS 9.3 for UNIX statistical software (SAS Institute, Gary, NC, USA).

### Ethics

The study was conducted according to the Nigerian National Code for Health Research Ethics. Ethical approval to conduct this study was obtained from the Institute of Human Virology Nigeria health research ethics committee and the University of Maryland School of Medicine Institutional Review Board. Written informed consent was obtained from all participants before enrollment in the study.

## Results

The mean (SD) age of the participants was 38 (8) years while their mean (SD) body mass index (BMI [kg/m^2^]) was 27 (5). Most (57%, 111/194) of the participants were married and premenopausal (83%, 161/194). The characteristics of the study participants at baseline are shown in Table 1. Participants returned for follow-up visits at a median (IQR) time of 5.7 (5.4–7.5) months.

**Table 1:** Baseline characteristics of women in the study population.

	All participants, *n* = 194	HIV positive, *n* = 139	HIV negative, *n* = 55

		Mean (SD)	
Characteristics			
Age, years	38 (8)	38 (8)	38 (9)
Body mass index, kg/m^2^	27 (5)	26 (4)	29 (6)
Age at sexual initiation, years	19 (3)	19 (3)	20 (4)
Total sex partners	4 (3)	4 (3)	3 (2)

		**n (%)**	

Marital status			
Married	111 (57)	72 (52)	39 (71)
Not married	83 (43)	67 (48)	16 (29)
Education, years completed			
≤6 years	23 (12)	17 (12)	6 (11)
7–12	125 (64)	100 (72)	25 (45)
>12	46 (24)	22 (16)	24 (44)
Contraceptive use	75 (37)	46 (33)	29 (53)
Condom use	1 (0.5)	1 (0.7)	0 (0)
Douching	129 (66)	96 (69)	33 (60)
Menopausal status			
Pre-menopausal	161 (83)	117 (85)	44 (80)
Post-menopausal	32 (17)	21 (15)	11 (20)

**Figure 1:**
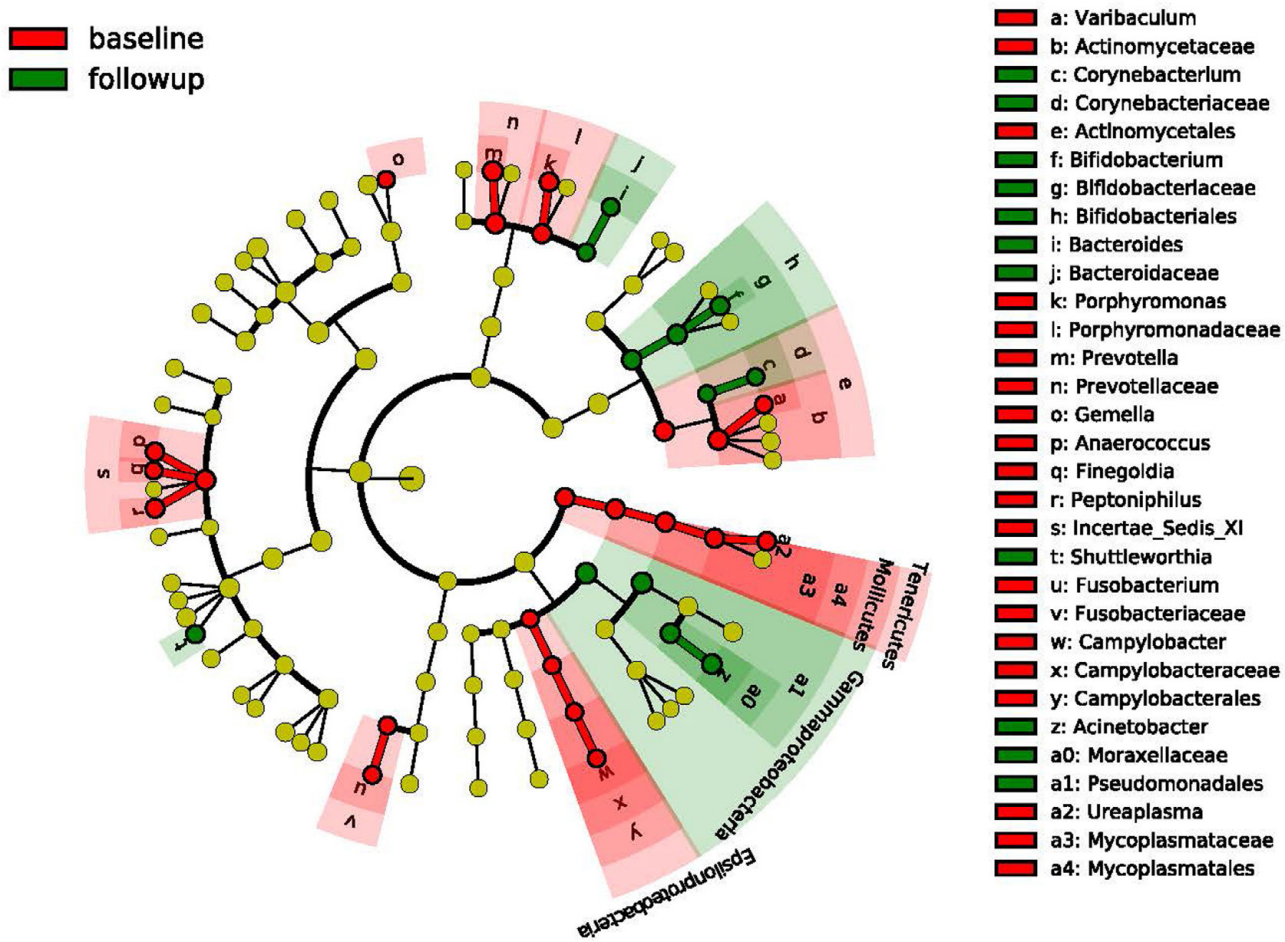
Cladogram representing the taxonomic hierarchical structure of the identified phylotype biomarkers. Each filled circles represents one biomarker; red, phylotypes statistically overrepresented at baseline; green, phylotypes overrepresented at follow-up. The diameter of a circle is proportional to the phylotype’s relative abundance; phylum and class are marked in their names on the Cladogram; and the order, family, or genera are named in alphabets and labeled in legend.

**Figure 2:**
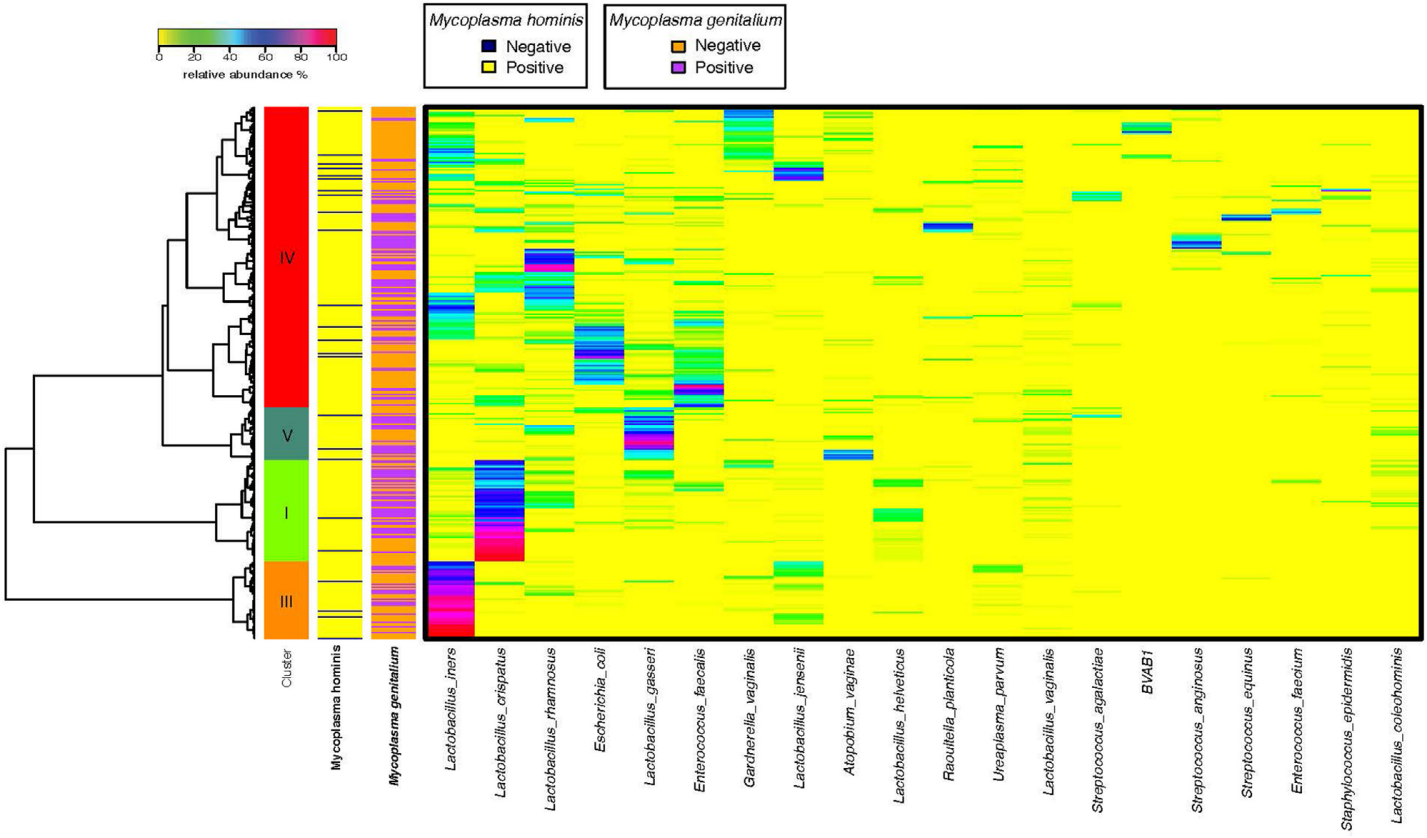
Community survey of vaginal microbiota samples collected from 194 female participants enrolled in this study. Relative abundance of the 20 most abundant phylotypes are shown in heatmap. Ward linkage clustering is used to clusters samples based on their Jensen–Shannon distance calculated in vegan package in R (29). Identified community state types (CSTs) are labeled as I, III, and IV, according to the previous naming convention (34).

We obtained mid-vaginal samples at baseline and at follow-up visits from 194 participants yielding 388 samples from which we generated a total of 12.9 million high quality reads with about 36,609 reads per sample. We identified 122 unique operational taxonomic units (OTUs) in this study population. Figure 1 shows the taxonomic hierarchical structure of the identified phylotype biomarkers. We observed four community state types (CSTs) of vaginal microbiota based on community composition and structure. Three of these CSTs were dominated by *Lactobacillus crispatus, L. iners*, and *L. gasseri*, while the fourth comprised a diverse set of strict and facultative anaerobes. Figure 2 shows the top 20 most abundant bacterial taxa, and *M. genitalium* and *M. hominis* in the vaginal microbiota.

At baseline, 72% (139/194) of the women were positive for HIV, 30% (43/142) for *M. genitalium*, 45% (87/194) for *M. hominis*, and 40% (77/194) for hrHPV. Fewer women (16%, 23/142) were persistently positive for *M. genitalium*, 30% (59/194) for *M. hominis*, and 31% (60/194) for hrHPV. The distribution of *Mycoplasma* spp. and hrHPV in the study population in total and by HIV status is shown in Table 2. HIV positive women were significantly positive for persistent *M. genitalium* (*p* < 0.001), *M. hominis* (*p* < 0.001), and hrHPV (*p* < 0.01) compared to HIV negative women. HPV52 (10%, 20/194) and HPV35 (9%, 17/194) were the most common persistent hrHPV infections in the study population (Table 3). HIV infection was positively associated with prevalent hrHPV (OR 2.34, 95% CI 1.13–5.10, *p* 0.01) and persistent high-risk HPV infections (OR 2.51, 95% CI 1.12–6.09, *p* 0.02), Table 4.

**Table 2:** Distribution of mycoplasma and high-risk human papillomavirus (hrHPV) in the study population in total and by HIV status.

	All participants	HIV positive	HIV negative	*p*-Value
*M. genitalium*	*n* = 142	*n* = 101	*n* = 41	
Prevalence	43 (30.3)	28 (27.7)	15 (36.6)	0.31
Persistence	23 (16.2)	23 (22.7)	0 (0)	<0.001
*M. hominis*	*n* = 194	*n* = 139	*n* = 55	
Prevalence	87 (44.8)	48 (34.5)	39 (70.1)	<0.001
Persistence	59 (30.4)	58 (41.7)	1 (1.7)	<0.001
hrHPV	*n* = 194	*n* = 139	*n* = 55	
Prevalence	77 (39.7)	63 (45.3)	14 (25.4)	0.01
Persistence	60 (30.9)	50 (35.9)	10 (18.2)	0.01

**Table 3:** High-risk human papillomavirus (hrHPV) types among the 194 study participants, in decreasing order of prevalence.

HPV types	Prevalent hrHPV *n* (%)	Persistent hrHPV *n* (%)
HPV 52	23 (11.9)	20 (10.3)
HPV 35	22 (11.3)	17 (8.8)
HPV 18	9 (4.6)	4 (2.1)
HPV 31	9 (4.6)	8 (4.1)
HPV 33	8 (4.1)	3 (1.6)
HPV 51	7 (3.6)	5 (2.6)
HPV 16	6 (3.1)	4 (2.1)
HPV 56	5 (2.6)	2 (1.0)
HPV 59	5 (2.6)	1 (0.5)
HPV 58	4 (2.1)	3 (1.6)
HPV 45	4 (2.1)	3 (1.6)
HPV 39	3 (1.6)	2 (1.0)

**Table 4:** Association between potential risk factors and high-risk human papillomavirus (hrHPV) infections.

Variable	Total *n*	Prevalent hrHPV			Persistent hrHPV		
		*n* (%)	OR (95% CI)	*p*-Value	*n* (%)	OR (95% CI)	*p*-Value
**Age, years**							
<30	19	6 (31)	0.59 (0.14–2.17)	0.55	4 (21)	0.64 (0.12–2.77)	0.74
30 to <45	141	55 (39)	0.81 (0.33–1.87)	0.72	46 (32)	1.16 (0.48–2.95)	0.88
≥45	34	15 (44)	Ref. (1.00)		10 (29)	Ref. (1.00)	
**Body mass index, kg/m^2^**							
Normal weight, 18.5 to <25	62	31 (50)	1.98 (0.85–4.71)	0.11	24 (39)	2.38 (0.94–6.38)	0.06
Overweight, 25 to <30	75	24 (32)	0.94 (0.40–2.20)	1.00	22 (29)	1.57 (0.62–4.17)	0.40
Obese, ≥30	48	16 (33)	Ref. (1.00)		10 (21)	Ref. (1.00)	
**Marital status**							
Married	111	44 (40)	1.04 (0.56–1.95)	0.99	35 (31)	1.06 (0.55–2.08)	0.95
Not married	83	32 (38)	Ref. (1.00)		25 (30)	Ref. (1.00)	
**Education, years completed**							
≤6 years	23	14 (61)	2.61 (0.84–8.49)	0.10	9 (39)	1.46 (0.44–4.69)	0.64
7–12	125	45 (36)	0.96 (0.45–2.07)	1.00	37 (20)	0.96 (0.43–2.18)	1.00
>12	46	17 (37)	Ref. (1.00)		14 (30)	Ref. (1.00)	
**Age at sexual initiation, years**							
≤19	98	41 (42)	1.61 (0.59–4.17)	0.42	33 (34)	1.14 (0.41–3.36)	0.97
19–22	67	26 (39)	1.42 (0.49–4.35)	0.63	19 (28)	0.89 (0.30–2.78)	1.00
>22	26	8 (31)	Ref. (1.00)		8 (31)	Ref. (1.00)	
**Total lifetime sex partners**							
1	31	8 (26)	0.62 (0.20–1.80)	0.48	7 (23)	0.61 (0.18–1.85)	0.49
2–4	106	48 (45)	1.48 (0.72–3.08)	0.31	35 (33)	1.04 (0.49–2.22)	1.00
≥5	56	20 (36)	Ref. (1.00)		18 (32)	Ref. (1.00)	
**Vaginal pH**							
<4.5	9	1 (11)	0.20 (0.04–1.56)	0.18	0 (0)	–	
4.5 to <5.5	13	9 (69)	3.58 (0.95–16.6)	0.06	9 (69)	5.28 (1.39–24.5)	0.01
≥5.5	172	66 (38)	Ref. (1.00)		51 (30)	Ref. (1.00)	
**HIV status**							
Positive	139	62 (45)	2.34 (1.13–5.10)	0.01	50 (36)	2.51 (1.12–6.09)	0.02
Negative	55	14 (25)	Ref. (1.00)		10 (18)	Ref. (1.00)	

*Table shows exact odds ratios (ORs) and 95% confidence intervals (CIs) based on logistic regression models*.

*n, number of women*.

We examined several variables for associations with *M. genitalium* and *M. hominis* (Tables 5 and 6). HIV infection was significantly associated with prevalent and persistent *M. hominis*. Given that HIV was also associated with hrHPV infections, we adjusted for this variable in multivariate models examining the associations between *M. genitalium* and *M. hominis*, and hrHPV infections. In the age adjusted analyses, the association between persistent *M. hominis* and persistent hrHPV was statistically significant (OR 11.1, 95% CI 2.37–51.6, *p* 0.002). This association remained statistically significant, after further adjustment for HIV status (OR 8.78, 95% CI 1.49–51.6, *p*-value 0.01) Table 7. Among the women, we also observed that there was significant association between HIV and prevalent *M. hominis* positivity, and prevalent hrHPV (OR 5.53, 95% CI 1.08–55.2, *p* 0.03), compared to HIV and prevalent *M. hominis* negative women. Similarly, there was significant association between HIV and persistent *M. hominis* positivity, and persistent hrHPV infections (OR 3.28, 95% CI 1.31–8.74, *p* 0.008), compared to HIV and persistent *M. hominis* negative women. There was no significant association between being positive for HIV and prevalent or persistent *M. genitalium*, and prevalent or persistent hrHPV infection.

**Table 5:** Association between sociodemographic characteristics and potential risk factors for *Mycoplasma genitalium*.

Variable	Total *n* (*n* = 142)	Prevalent *M. genitalium*			Persistent *M. genitalium*		
		*n* (%)	OR (95% CI)	*p*-Value		OR (95% CI)	*p*-Value
**Age, years**							
<30	13	3 (23)	0.77 (0.10–4.44)	1.00	3 (23)	0.76 (0.19–24.7)	0.75
30 to <45	104	33 (32)	1.19 (0.42–3.72)	0.91	17 (16)	1.68 (0.35–10.9)	0.71
≥45	25	7 (28)	Ref. (1.00)		3 (12)	Ref. (1.00)	
**Body mass index, kg/m^2^**							
Normal weight, 18.5 to <25	45	17 (38)	1.60 (0.55–4.90)	0.46	6 (13)	1.84 (0.34–11.1)	0.63
Overweight, 25 to <30	61	16 (26)	0.94 (0.33–2.82)	1.00	12 (20)	2.93 (0.68–15.4)	0.18
Obese, ≥30	33	9 (27)	Ref. (1.00)		4 (12)	Ref. (1.00)	
**Marital status**							
Married	84	21 (25)	0.58 (0.24–1.20)	0.14	16 (19)	1.48 (0.44–5.21)	0.64
Not married	58	22 (38)	Ref. (1.00)		7 (12)	Ref. (1.00)	
**Education, years completed**							
≤6 years	14	4 (28)	0.68 (0.12–3.01)	0.82	0 (0)	-	
7–12	93	26 (28)	0.65 (0.26–1.64)	0.42	19 (20)	2.91 (0.75–14.1)	0.14
>12	35	13 (37)	Ref. (1.00)		4 (11)	Ref. (1.00)	
**Ageat sexual initiation, years**							
≤19	75	24 (32)	1.01 (0.31–3.68)	1.00	14 (19)	8.23 (0.93–72.4)	0.06
19–22	47	13 (28)	0.83 (0.23–3.25)	0.97	8 (17)	5.00 (0.54–46.2)	0.15
>22	19	6 (31)	Ref. (1.00)		1 (5)	Ref. (1.00)	
**Total lifetime sex partners**							
1	21	5 (24)	0.49 (0.12–1.73)	0.35	3 (14)	0.55 (0.07–3.18)	0.71
2–4	72	19 (36)	0.59 (0.24–1.32)	0.21	13 (18)	1.43 (0.39–5.47)	0.74
≥5	49	19 (39)	Ref. (1.00)		7 (14)	Ref. (1.00)	
**Vaginal pH**							
<4.5	3	0 (0)	–	–	2 (67)	3.90 (0.33–45.6)	0.27
4.5 to <5.5	9	1 (11)	0.24 (0.01–2.07)	0.34	1 (11)	0.65 (0.06–6.65)	0.71
≥5.5	130	42 (32)	Ref. (1.00)		20 (15)	Ref. (1.00)	
**HIV status**							
Yes	101	28 (28)	0.66 (0.28–1.56)	0.39	23 (23)	–	
No	41	15 (36)	Ref. (1.00)		0 (0)	Ref. (1.00)	

*Table shows exact odds ratios (ORs) and 95% confidence intervals (CIs) based on logistic regression models; *n* = number of women*.

**Table 6:** Association between sociodemographic characteristics and potential risk factors for *Mycoplasma hominis*.

Variable		Prevalent *M. hominis*			Persistent *M. hominis*		
	Total *n* (*n* = 194)	*n* (%)	OR (95% CI)	*p*-Value		OR (95% CI)	*p*-Value
**Age, years**							
<30	19	7 (37)	0.74 (0.19–2.67)	0.82	7 (37)	2.98 (0.39–37.7)	0.41
30 to <45	141	65 (46)	1.08 (0.47–2.49)	0.98	43 (30)	1.80 (0.52–6.16)	0.41
≥45	34	15 (44)	Ref. (1.00)		9 (26)	Ref. (1.00)	
**Body mass index, kg/m^2^**							
Normal weight, 18.5 to <25	62	30 (48)	1.01 (0.44–2.31)	1.00	18 (29)	4.17 (1.01–19.9)	0.04
Overweight, 25 to <30	75	32 (43)	0.81 (0.36–1.78)	0.69	29 (39)	3.40 (1.01–12.1)	0.04
Obese, ≥30	48	23 (48)	Ref. (1.00)		10 (21)	Ref. (1.00)	
**Marital status**							
Married	111	47 (42)	0.79 (0.42–1.45)	0.50	35 (31)	0.80 (0.29–2.14)	0.80
Not married	83	40 (48)	Ref. (1.00)		24 (29)	Ref. (1.00)	
**Education, years completed**							
≤6 years	23	8 (35)	0.41 (0.12–1.29)	0.14	8 (35)	2.57 (0.47–15.7)	0.36
7–12	125	53 (42)	0.56 (0.26–1.18)	0.14	45 (36)	4.58 (1.27–18.0)	0.01
>12	46	26 (56)	Ref. (1.00)		6 (13)	Ref. (1.00)	
**Age at sexual initiation, years**							
≤19	98	42 (43)	0.47 (0.17–1.23)	0.13	35 (36)	3.25 (0.48–25.1)	0.28
19–22	67	29 (43)	0.48 (0.16–1.32)	0.17	20 (30)	2.17 (0.30–17.5)	0.58
>22	26	16 (61)	Ref. (1.00)		3 (11)	Ref. (1.00)	
**Total lifetime sex partners**							
1	31	17 (55)	0.91 (0.34–2.42)	1.00	5 (16)	0.30 (0.05–1.55)	0.18
2–4	106	38 (36)	0.42 (0.20–0.85)	0.01	37 (35)	0.95 (0.27–3.03)	1.00
≥5	56	32 (57)	Ref. (1.00)		17 (30)	Ref. (1.00)	
**Vaginal pH**							
<4.5	9	1 (11)	0.13 (0.01–1.12)	0.06	2 (22)	0.14 (0.01–0.90)	0.03
4.5 to <5.5	13	4 (31)	0.48 (0.14–1.64)	0.24	4 (31)	0.86 (0.11–10.2)	1.00
≥5.5	172	82 (48)	Ref. (1.00)		53 (31)	Ref. (1.00)	
**HIV status**							
Yes	139	48 (34)	0.21 (0.10–0.44)	<0.001	58 (42)	51.6 (6.99–>999)	<0.001
No	55	39 (71)	Ref. (1.00)		1 (2)	Ref. (1.00)	

*Table shows exact odds ratios (ORs) and 95% confidence intervals (CIs) based on logistic regression models; *n* = number of women*.

**Table 7:** Multivariate association between *M. genitalium* and *M. hominis*, and high-risk human papillomavirus (hrHPV) infections.

	*N* positive/negative	Exact odds ratio (95% CI)	*p*-Value	Exact odds ratio (95% CI)	*p*-Value

		Model 1		Model 2	

			Prevalent hrHPV		
Prevalent *M. genitalium*	15/45				
Positive		0.65 (0.28–1.44)	0.33	0.68 (0.29–1.53)	0.41
Negative		Ref. (1.00)		Ref. (1.00)	
Prevalent *M. hominis*	34/42				
Positive		0.99 (0.53–1.84)	1.00	1.35 (0.66–2.56)	0.49
Negative		Ref. (1.00)		Ref. (1.00)	

			**Persistent hrHPV**		

Persistent *M. genitalium*	7/44				
Positive		0.80 (0.22–2.64)	0.90	0.43 (0.10–1.87)	0.32
Negative		Ref. (1.00)		Ref. (1.00)	
Persistent *M. hominis*	25/35				
Positive		11.1 (2.37–51.6)	0.002	8.78 (1.49–51.6)	0.01
Negative		Ref. (1.00)		Ref. (1.00)	

*Table shows odds ratios (ORs) and 95% confidence intervals (CIs) for women with prevalent or persistent *M. genitalium* and *M. hominis*, compared to women who were negative. The ORs and 95% CI were based on exact logistic regression models*.

*n = number of women with hrHPV infection, who were positive or negative for M. genitalium and M. hominis*.

*Model 1 = age-adjusted*.

*Model 2 = model 1 + HIV status (positive vs. negative)*.

## Discussion

This is the first study to examine associations between persistent *M. genitalium* and *M. hominis* in the vaginal microbiota and prevalent as well as persistent hrHPV infections to our knowledge. We found high prevalence and persistence of *M. hominis* and *M. genitalium* in the vaginal microbiota of our study participants. There was significant association between persistent *M. hominis* and persistent hrHPV infections independent of HIV status.

Previous studies of the prevalence of cervico-vaginal *Mycoplasma* spp. in African women include studies of commercial sex workers in Kenya where the prevalence of *M. genitalium* was 13% and in Niger Republic where the prevalence of *M. hominis* was 37% (35, 36). Another study of *M. hominis* among HIV-positive women found a prevalence of 16.7% (37). Direct comparison of these results with ours is limited by differences in the study populations and method of characterizing *Mycoplasma* spp. While we used sequence analysis of V3–V4 hypervariable region of the 16S rRNA gene, which samples only a fraction of the amplicons, and is less sensitive compared to PCR, the other studies used PCR, ELISA, or molecular hybridization to identify and characterize *Mycoplasma* spp. Some of the primers used in these methods do not react with all *Mycoplasma spp*. strains (38).

In the present study, we did not observe significant associations between prevalent *Mycoplasma* spp. and prevalent hrHPV infection. The results of previous studies on the association between prevalent *M. genitalium, M. hominis*, and hrHPV infections have been contradictory (6, 8, 9). Consistent with our findings, no significant associations were observed between prevalent *M. genitalium* or *M. hominis* and prevalent hrHPV infection in studies conducted among Brazilian (8) and South Korean (9) women. Contrary to our results, Biernat-Sudolska et al. reported positive associations between prevalent *M. genitalium* or *M. hominis* and prevalent hrHPV infection among women (6). The inconsistence of these studies may be due to inherent differences in the populations studied and the methodologies used. In the present study, we also observed significant associations between persistent *M. hominis* and persistent hrHPV infections. As similar studies on the association between persistence of *Mycoplasma* spp. and persistent hrHPV infection have not yet been conducted elsewhere to the best our knowledge, we were unable to compare these findings.

The mechanism by which *Mycoplasma* spp. may facilitate hrHPV infection or cervical carcinogenesis remains unclear. Like other sexually transmitted genital pathogens, *Mycoplasma* spp. may be associated with changes in epithelial cells that facilitate entry of HPV virions or with changes in the immunological response pathways that decrease the host’s ability to resolve HPV infection (39, 40). Several bacteria, particularly those capable of establishing persistent infections, can alter host cell cycles, affect apoptotic pathways, and stimulate the production of inflammatory substances linked to DNA damage, thus potentially promote abnormal cell growth (12).

Cervical somatic cells’ methylation is another potential pathway through which *Mycoplasma* spp. can be associated with cervical carcinogenesis (41–47). Several *Mycoplasma* species infect eukaryotic cells, where they activate genes and have been associated with malignant transformations (14, 17, 48, 49). Recently, it was shown that upon cellular infection, CG- and GATC-specific DNA cytosine methyltransferases from some *Mycoplasma* spp. efficiently translocate to the nucleus and methylate several regions of the genome (50). This dramatic and chaotic modification of the somatic epigenetic cellular landscape likely results in a disorganized pattern of gene expression. Zhang et al. demonstrated that infections of HPV E6- and E7-immortalized cervical cells by some *Mycoplasma* spp., including *M. genitalium* and *M. hominis*, were associated with profound alterations in cytokines’ gene expression (19). They concluded that chronic and persistent infection with these seemingly low-virulence mycoplasmas could gradually but significantly affect many important biological characteristics of mammalian cells and lead to malignant transformation (14, 15, 51).

Little is known about the factors associated with presence of *Mycoplasma* spp. in the female genitalia. HIV positive women in our study were more likely to be positive for both *M. genitalium* and *M. hominis*. However, other indicators of risky sexual behavior, including earlier age at sexual initiation and number of lifetime sexual partners, were not associated between *M. genitalium* and *M. hominis*, and either vaginal *M. genitalium* or *M. hominis* in this study. We did not observe significant associations between *M. genitalium* and *M. hominis*, and sociodemographic variables including participants’ age, marital status, and level of education. These results are consistent with a previous study conducted among Kenyan women (35). The Kenyan study of female sex workers also reported that using condoms most or all the time was more likely to be associated with presence of *M. genitalium* (35), while sexual intercourse during menstruation was inversely associated with *M. genitalium*. Given that the level of condom use in our study population was very low, we were unable to examine its relation to *M. genitalium* and *M. hominis* positivity.

Our study has several important limitations. We identified *M. genitalium* and *M. hominis* in the vaginal microbiota by sequencing the V3–V4 hypervariable regions of the 16S rRNA gene, this method has not been clinically validated as a test for *Mycoplasma* spp. As sequencing the 16S rRNA gene is a sampling approach, the power to detect *M. genitalium* or *M. hominis* is correlated with the depth of sequencing and limited to *x* logs if 10*^x^* reads are sequenced per sample. Also, our sample size was small, as indicated by the wide confidence intervals. Therefore, we advise that our results be interpreted with caution and recommend additional studies with larger sample sizes should be conducted. Because we evaluated *M. genitalium* and *M. hominis*, and hrHPV infections at the same time, we are unable to rule out reverse causation.

In conclusion, our study shows significant associations between persistent *M. hominis* in the vaginal microbiota and persistent hrHPV infections. These findings suggest that *M. hominis* and possibly, other mycoplasmas may play a role in hrHPV induced cervical carcinogenesis and warrant further studies. If confirmed, testing and treating for *M. hominis* and possibly, other mycoplasma in women with hrHPV infections may preempt hrHPV persistence and help alleviate the burden of cervical cancer.

## Author Contributions

SA conducted the data analyses, interpreted the data, and drafted the manuscript. CA designed and obtained funding for the study. AF processed the samples, performed biochemical assays, and collated the laboratory data. BM, DZ, JR, and CA contributed to the data analysis and interpretation. Each author approved the final version of the manuscript and agreed to be accountable for all aspects of the work.

## Conflict of Interest Statement

The authors declare that the research was conducted in the absence of any commercial or financial relationships that could be construed as a potential conflict of interest.
